# Primary Cardiac Tumors: Clinical Presentations and Pathological Features in a Multicenter Cohort

**DOI:** 10.3390/diagnostics15151951

**Published:** 2025-08-04

**Authors:** Esra Çobankent Aytekin, Kemal Behzatoğlu, Arzu Akçay, Ayşe Özgün Şahin, Naile Kökbudak, Fahriye Kılınç, Aylin Okçu Heper, Olcay Kurtulan, Gülay Özbilim, Reyhan Eğilmez, Tülay Koç, Doğuş Özdemir Kara, Elif Ocak, Ali Aköz, İrem Hicran Özbudak

**Affiliations:** 1 Department of Pathology, Konya Numune State Hospital, 42000 Konya, Turkey; esracobankent@hotmail.com; 2 Department of Pathology, School of Medicine, Acıbadem University, 34752 Istanbul, Turkey; kbehzatoglu@hotmail.com; 3 Acıbadem Pathology Group, Department of Pathology, Yeni Yüzyıl University, 34010 Istanbul, Turkey; arzuakcay12@gmail.com; 4 Council of Forensic Medicine, 34180 Istanbul, Turkey; aozgunsahin@gmail.com; 5 Department of Pathology, Faculty of Medicine, Necmettin Erbakan University, 42080 Konya, Turkey; naileyaldiz@hotmail.com (N.K.); drfahriyek@gmail.com (F.K.); 6 Department of Pathology, Faculty of Medicine, Ankara University, 06230 Ankara, Turkey; aylinheper@yahoo.com; 7 Department of Pathology, Faculty of Medicine, Hacettepe University, 06100 Ankara, Turkey; olcaykurtulan@gmail.com; 8 Department of Pathology, Faculty of Medicine, Akdeniz University, 07070 Antalya, Turkey; gulayozbilim@hotmail.com; 9 Department of Pathology, Faculty of Medicine, Cumhuriyet University, 58140 Sivas, Turkey; egilmezreyhan@gmail.com (R.E.); tkoc@cumhuriyet.edu.tr (T.K.); 10 Council of Forensic Medicine, 06520 Ankara, Turkey; dogusdr@yahoo.com; 11 Department of Pathology, Van Training and Research Hospital, 65000 Van, Turkey; elf.ocak@gmail.com; 12 Department of Public Health, Konya Provincial Health Directorate, 42000 Konya, Turkey; dr.akoz.ali@gmail.com

**Keywords:** cardiac neoplasm, myxoma, papillary fibroelastoma, metastatic tumor

## Abstract

**Background:** Cardiac tumors are rare neoplasms with a wide spectrum of clinical presentations, ranging from asymptomatic cases to fatal outcomes. According to the 2021 thoracic tumor classification of the World Health Organization (WHO), papillary fibroelastoma (PFE) is the most common primary cardiac tumor. This study aimed to aggregate and examine data regarding the prevalence, clinical characteristics, and histological results of cardiac tumors. **Methods:** This multicenter retrospective study was conducted across seven tertiary care institutions and included 274 patients diagnosed with histopathologically confirmed cardiac tumors between January 2013 and December 2024. **Results:** This study included 274 patients, with an average age of 52.6 ± 16.6 years. Of the study participants, 120 (43.8%) were male and 154 (56.2%) were female. The most prevalent clinical manifestations were dyspnea (43.7%), thoracic pain (22.5%), and cardiac palpitations (21.1%). Echocardiography was the principal diagnostic method, revealing an average tumor size of 3 cm. The most commonly observed mass was cardiac myxoma (CM) in 192 patients (70.1%). The second most frequently detected mass was PFE (28 cases, 10.2%). The third most common cardiac mass was a metastatic tumor (6.9%). Surgical resection was performed in all patients, with infection being the most prevalent consequence, followed by effusion. **Conclusions:** Cardiac tumors, albeit uncommon, provide considerable diagnostic and treatment difficulties. Our research is founded on an extensive case series that has been histopathologically validated and sourced from various national tertiary centers. This comprehensive dataset offers epidemiological and clinical insights regarding heart tumors in Turkey. Another key finding of our study is that, even though the 5th edition of the 2021 WHO Classification of Thoracic Tumors lists PFE as the most common primary cardiac tumor, myxoma is actually the most common primary cardiac tumor in our study and in many other studies. This finding demonstrates a significant discrepancy between the current international classification and real-world data and suggests that tumor distribution may be related to regional and demographic differences.

## 1. Introduction

Primary cardiac neoplasms are exceptionally rare, accounting for less than 0.03% of the cases identified in autopsy-based investigations. Approximately 75% of primary tumors are benign, while approximately 25% are malignant [1]. Secondary cardiac tumors (SCTs) that result from metastasis to the heart are observed significantly more often than PCTs [2]. Papillary fibroelastomas (PFEs) are the most commonly identified subtype according to the updated World Health Organization (WHO) classification of thoracic neoplasms [3].

Cardiac tumors can arise from any anatomical compartment of the heart, including the endocardial surface, myocardium, pericardium, or valvular structures, each of which has distinct clinical implications. Clinical manifestations are highly variable, ranging from incidental findings in asymptomatic individuals to symptoms such as exertional dyspnea, arrhythmias, embolic strokes, or constitutional signs, including weight loss and fatigue. These tumors may mimic common cardiovascular pathologies, leading to diagnostic delays [4,5].

Diagnostic pathways typically involve a combination of imaging modalities. Transthoracic and transesophageal echocardiography serves as a first-line tool for initial detection, whereas cardiac magnetic resonance imaging (MRI) and computed tomography (CT) contribute to further anatomical characterization and surgical planning. Nevertheless, a definitive diagnosis requires histopathological confirmation after surgical excision or biopsy [5].

Surgical resection remains the primary therapeutic approach for resectable cardiac tumors, regardless of their histological behavior. However, treatment strategies must be tailored to the tumor’s anatomical location, size, malignant potential, and functional status. Complete resection and avoidance of critical intracardiac structural damage are key determinants of postoperative prognosis.

This study provides a comprehensive multicenter analysis of cardiac tumors in Turkey, focusing on their epidemiological patterns, clinical profiles, and histopathological spectrum. By integrating data from multiple tertiary care institutions over a twelve-year period, this study aimed to contribute valuable insights into the diagnosis and management of both benign and malignant cardiac tumors in clinical practice.

## 2. Materials and Methods

### 2.1. Study Design and Setting

This investigation was structured as a multicenter retrospective analysis encompassing data from seven tertiary referral institutions across Turkey. The study cohort comprised 274 patients who underwent surgical excision of cardiac tumors, with histopathological confirmation serving as the inclusion criterion. Cases were identified from institutional archives from between January 2013 and December 2024. Ethical clearance was obtained from the Institutional Review Boards (IRBs) of all participating centers, and the research was conducted in adherence to internationally accepted ethical principles governing human subject research.

### 2.2. Data Acquisition and Variables

Clinical, demographic, and pathological data were retrospectively extracted from the medical records. Variables of interest included age, sex, symptomatology at presentation, imaging modalities used, tumor localization and size, histopathological diagnosis, treatment modality, and short-term postoperative outcomes. The dataset was compiled into a centralized anonymized database for uniform analysis across the centers.

### 2.3. Histopathological Evaluation

Resected tumor specimens were examined in the designated institutional pathology departments. After macroscopic assessment, samples were fixed in formalin and processed using standard histological protocols. Hematoxylin and eosin (H&E) staining was employed as the primary diagnostic tool, supplemented by histochemical and immunohistochemical markers, when further tissue characterization was warranted. Tumor classification followed the criteria established in the fifth edition of the WHO classification of thoracic tumors, ensuring consistency with international diagnostic standards [3].

### 2.4. Ethical Considerations

This study was conducted in accordance with the Declaration of Helsinki and adhered to national and institutional standards for human research. Ethical approval was granted by the Medical Scientific Research Ethics Committee of Akdeniz University. The committee’s approval (decision number: TBAEK-123), obtained on 30 January 2025, confirmed that the proposed research, Clinical and Histopathological Analysis of Cardiac Tumors—A Multicenter Study in Turkey, met all scientific and ethical standards for medical research (ET0K).

### 2.5. Statistical Analysis

Descriptive findings are presented as numbers and percentages for categorical variables and as the mean ± standard deviation for continuous variables. Skewness and kurtosis parameters and their ratios to standard errors were compared for age and tumor size variables, and Kolmogorov–Smirnov tests were performed to determine the normal distribution. Independent samples t-tests were used to compare the means of continuous variables between two independent groups. A *p* value of <0.05 was considered statistically significant. Microsoft Excel and IBM SPSS v.22.0 were used for data cleaning, organization, and analysis.

## 3. Results

In total, 274 patients with cardiac tumors were included in this study. Age information was available for 272 of 274 patients. The mean age was 52.6 years (SD: 16.6), with 56.2% (154 females) and 43.8% (120 males) of patients being female and male, respectively. Clinical and imaging data were available for 32.4% of the patients (*n* = 274).

Asymptomatic presentation was observed in 23.9% of the patients. Most symptomatic patients exhibited cardiac symptoms (71.8%), with dyspnea being the most common symptom (43.7%). The other symptoms included chest pain (22.5%) and palpitations (16.9%). Embolic symptoms were present in 14.1% of patients, while systemic symptoms such as weight loss and weakness were reported in 21.1% of patients. High rates of post-diagnosis complications were noted, with infection (94.4%), effusion (84.5%), and arrhythmia (85.9%) being the most prevalent. Significant comorbid conditions included coronary artery disease (20.2%), diabetes mellitus (12.4%), and hypertension (11.2%). Echocardiography was the primary imaging modality used, providing initial diagnostic insights in 57.3% of cases (*n* = 89). The descriptive characteristics of the cardiac tumors are shown in Table 1.

**Figure 1:**
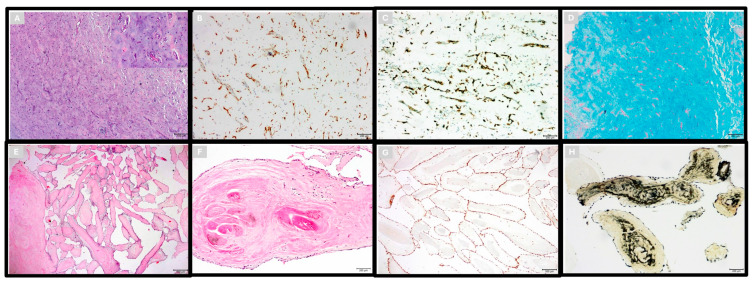
(**A**) Cardiac myxoma. Isolated mesenchymal cells with eosinophilic cytoplasm form cords and rings in myxoid background: H&E, ×200 and inset ×400. (**B**) Calretinin ×100. (**C**) CD34 ×100. (**D**) Myxoid stroma stained with Alcian Blue ×100. (**E**) PFE, with narrow, elongated, and branched papillary fronds composed of centrally avascular collagen: H&E ×40. (**F**) Avascular central layer composed of fibrous and elastin tissue: H&E ×100. (**G**) Vimentin ×100. (**H**) Elastic van Gieson ×100 [3].

The cardiac tumors were subjected to histopathological evaluation. Primary benign tumors were predominant, with myxomas being the most common, found in 192 cases (70.1%). Papillary fibroelastomas were the next most frequent benign tumors and were documented in 28 cases (10.2%) (Figure 1A–F). Other benign tumors, including vascular benign tumors and various unspecified types, were less common and were found in 5 (1.82%) and 11 (4.01%) cases, respectively.

**Figure 2:**
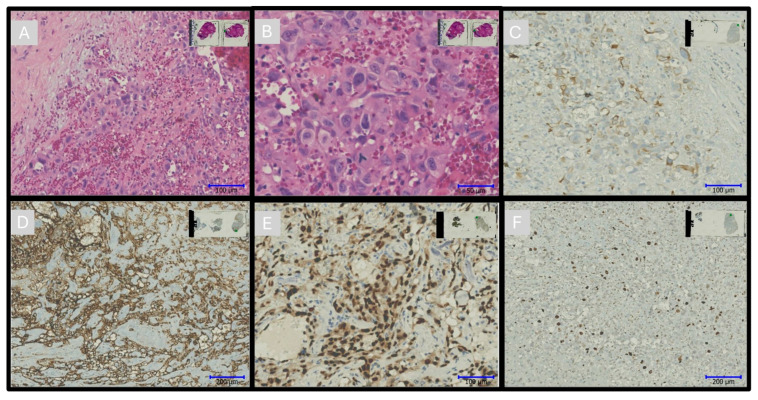
Epithelioid angiosarcoma. Angiosarcomas are characterized by vascular channels lined with atypical, pleomorphic endothelial cells. Areas of hemorrhage, necrosis, and mitotic figures are frequently observed. (**A**,**B**) H&E ×200, ×400. (**C**) Epithelioid angiosarcoma showing epithelioid cells positive for PanCK ×200. (**D**) Angiosarcomas consistently express vascular markers such as cluster of differentiation. CD31 ×100. (**E**) ERG ×200. (**F**) Ki67 ×100 [3].

Angiosarcomas were the most frequently observed malignant tumors and exhibited classic histopathological features, including atypical endothelial proliferation, extensive hemorrhage, and necrosis. Immunohistochemical staining demonstrated strong positivity for vascular markers such as CD31 and ERG, and high proliferative activity was confirmed with Ki-67 staining. Cytokeratin and synaptophysin were selectively positive in epithelial and neuroendocrine subtypes. The histopathological spectrum included sarcomas, vascular malignancies, and hematolymphoid tumors, each showing distinct cellular architecture and immunophenotypic profiles that supported differential diagnosis. The morphological characteristics of primary malignant cardiac tumors are illustrated in Figure 2.

In the malignancy spectrum, primary malignant tumors comprised a small proportion of diagnoses. Sarcomas were identified in eight patients, accounting for 2.92% of cases, and vascular malignant tumors were noted in another five cases (1.82%) (Figure 2A–F). Other malignant tumors, including hematolymphoid malignancies and small-round-cell tumors, were found in six patients (2.2%). Metastatic tumors, indicative of secondary cancer spreading to the heart, were observed in 19 cases (6.93%). The distribution of cardiac tumor diagnoses is summarized in Table 2.

**Figure 3:**
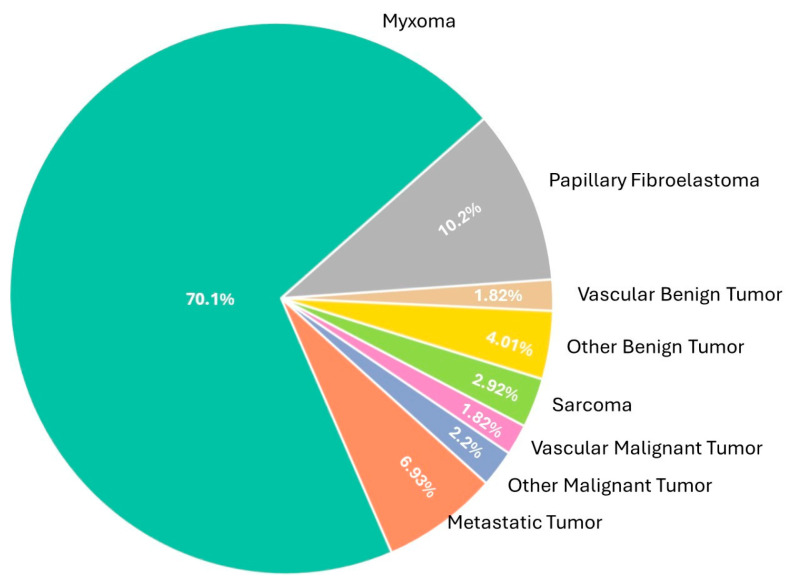
Pathological distribution of cardiac tumors.

The pathological classification of cardiac tumors in the cohort is visually summarized in Figure 3. Cardiac myxomas constituted the vast majority of cases (70.1%), followed by papillary fibroelastomas (10.2%). Metastatic cardiac tumors comprised 6.9% of the cohort, whereas sarcomas, vascular malignancies, and other benign tumors were less frequent, each accounting for 1.8–4.0% of cases (Figure 3).

This pie chart illustrates the relative frequencies of histopathologically confirmed cardiac tumor types in the study cohort (*n* = 274).

Data are presented as percentages based on absolute case counts of each tumor category. The most common tumor type was myxoma (70.1%), followed by papillary fibroelastoma (10.2%) and metastatic tumors (6.9%). Rare tumors such as sarcomas, vascular malignancies, and other benign variants were each observed in fewer than 5% of cases.

The left atrium was predominantly affected by myxomas, which constituted 98.5% (67 cases) of the tumors in this area. Although there was a more diverse pathology in the right atrium, the most common tumors were myxomas (50% of the cases). The mitral and tricuspid valves showed a predisposition for papillary fibroelastomas, comprising 60% and 80% of the tumors in these locations, respectively. In the left ventricle, the tumor types were split equally between papillary fibroelastoma and benign vascular tumors, each at 50%. The distribution of pathological diagnoses according to tumor location is shown in Table 3.

When stratified by symptom status, asymptomatic patients had a higher median age (62.0 years) compared to symptomatic patients (56.5 years), although this difference was not statistically significant (*p* = 0.23). Similarly, the mean tumor size was greater in asymptomatic patients (39.07 ± 19.69 mm) than in symptomatic patients (30.25 ± 20.57 mm), but without statistical significance (*p* = 0.14). The comparative analysis is presented in Table 4.

## 4. Discussion

PCTs represent a rare diagnostic category, with autopsy-based estimates placing their incidence below 0.33%. While the majority of these tumors are benign, their clinical implications remain substantial, given their propensity to cause hemodynamic obstruction, embolic events, or conduction abnormalities. Historically, CMs have been considered the most common PCTs, but recent WHO updates indicate that PFEs may now predominate. This discrepancy between current international classifications and real-world institutional data suggests that regional, demographic, or methodological factors may influence tumor distribution patterns [3,6,7,8,9,10].

This ten-year study looked at seven heart centers in Turkey. This adds important information to our knowledge. We found that 23.9% of the patients had no symptoms. This is important because it showed that these tumors can be hidden. Most patients with symptoms had trouble breathing (43.7%). This is often because the tumor is located in the left atrium, which can block the mitral valve. This blockage can cause fluid accumulation in the lungs or heart failure, leading to breathing problems. The other symptoms included heart palpitations (31.1%) and chest pain (22.5%). Some patients also had weight loss and weakness (21.1%), and some fainted due to embolic events (14.1%) [8,11,12,13].

The left atrium was the most common location for primary tumors, with 98.4% being CMs. Other studies have also shown that CMs are the most common PCTs, usually in the left atrium. The right atrium and pericardium were also common sites for myxomas and secondary tumors. Studies have shown that certain tumors prefer specific areas of the heart. PFEs are often present at valve sites, particularly the aortic valve [4,6,8,9,12,14]. Gecmen C. et al. found that the left atrium was the most common tumor site, followed by the mitral valves, with myxoma being the most common tumor [6]. In studies where PFE was the most common tumor, it was mostly found in a cardiac valve, especially the aortic valve. Many studies have reported the pericardium as the most common site for SCTs [7,12].

Rahouma et al. examined 8, 849 cases of heart tumors in 2020. It was found that 84.6% of these tumors were benign PCTs, of which 68.7% were myxomas. The study also noted that 9.7% were primary malignant cardiac tumors (PMCTs) and 4% were secondary tumors, showing a variety of cardiac tumors. Similarly, in our group, 85.8% of the tumors were benign and 70.1% were myxomas. PMCTs made up 7.3%, and secondary tumors comprised 6.9% [15]. Our study group had slightly more women, with an average age of 52.6 years, similarly to global trends in cardiac tumor patients.

CMs are the most common type of PCTs, particularly in women aged 40–60 years. Our results match this pattern, with most myxomas occurring randomly, less than 10% being familial, and them often being linked to conditions such as the Carney complex. Notably, patients with Carney complex-related myxomas tend to be younger and predominantly male, in contrast to the typical demographic, and these tumors show a higher propensity for recurrence [16,17].

In this study, the left atrium was the most common location of myxomas, which is consistent with the findings of other studies. This is because the structure and function of the LA may help tumors to grow or be found. The rate of silent cardiac myxomas varies greatly from 0% to 29% [16,18]. Our study found fewer silent cases, showing how these tumors can go unnoticed and how local practices affect their detection. Patients with Carney complex-related myxomas are often younger and more often male than those with sporadic myxomas. Recurrence is common in such cases [13,19]. In our study, two patients (3.4%) with the Carney complex had recurrences: a 56-year-old woman and a 55-year-old man.

It is widely agreed that the most common site for myxomas is the left atrium; however, there is variability in the literature regarding the second and third most common sites. In this study, the right atrium and interatrial septum were the second most common sites, which is in agreement with the results of other studies [16,18]. The rate of silent cardiac myxomas reported in the literature ranges from 0% to 29%. These numbers mostly come from single-center studies and can be affected by factors such as referral bias and local expertise. In our study, the rate of silent cardiac myxomas was 23.9%, which was consistent with the literature.

In our study, similarly to the literature, cardiac symptoms, especially dyspnea, were the most common [13,19].

PFEs are non-malignant cardiac tumors with fibrous and elastic growths. They are often found on the cardiac valves, especially the mitral and tricuspid valves. This matches other studies showing that these tumors prefer valves [6,7,9,10]. Tamin et al. examined 511 patients using pathology and echocardiography. It was found that PFEs are now the most common primary benign cardiac tumors, not myxomas, which was the old belief [9]. In this study, 112 patients had CM and 185 had PFEs. The study concluded that papillary fibroelastoma is the most common primary cardiac tumor [9].

Tamin et al.’s study noted that myxomas were thought to be the most common benign cardiac tumors in adults because most data came from autopsies. Our study included 19 autopsy cases, of which secondary tumors (13 cases) were the most common, and myxomas (4 cases) were the most common primary tumors (6 cases). In our study, myxomas (192 cases) were approximately seven times more common than PFEs (28 cases). A 2021 study by Li et al., which did not include autopsies, found myxomas to be the most common primary cardiac tumors in 94.8% of 225 patients [8]. Most studies have reported similar findings [6,7,15].

In our study, similarly to previous studies, PFEs were most frequently observed in the heart valves, with a higher prevalence in the mitral and tricuspid valves. Although the literature commonly reports that the aortic valve is the most frequent location for PFEs [6,7,9,10], studies have also identified the mitral valve [20] as the most common site. The echocardiographic appearance of myxomas and/or papillary fibroelastomas is often highly characteristic. However, it is crucial to distinguish these from valvular vegetation and atrial thrombi. Therefore, studies incorporating histopathological diagnoses, as in the present study, are of significant importance in the medical literature.

All adipocytic tumors in the benign tumor group (three lipomas, one lipomatous hypertrophy of the atrial septum, and one hibernoma) occurred in elderly female patients. These rare tumors were more frequent in this study than in previous studies [6,7]. Among the other benign tumors, there were three fibromas, one rhabdomyoma, one cystic tumor of the atrioventricular (A-V) node, and a very rare cardiac glomus tumor [21].

Regarding malignant cardiac neoplasms, PMCTs such as sarcomas, especially angiosarcomas, represent a smaller yet significant portion of our findings. These tumors are particularly aggressive and have a dismal prognosis, often involving the right atrium and resulting in complications such as pericardial effusion and cardiac tamponade [22,23]. In our study, PMCTs were found in 7.3% of cases, with a notable presence of undifferentiated sarcomas, suggesting variance from broader literature trends [6,7,15].

SCTs, which are more prevalent than PCTs, typically arise from the metastatic spread of primary malignancies located elsewhere, such as in the breasts, lungs, or esophagus. The prevalence of SCTs in our study was consistent with broader epidemiological data, yet it underscores the critical need for comprehensive oncological assessments in patients with known malignancies [4,24]. Secondary tumors were seen less frequently in our study (6.9%) compared to the literature and lung cancer metastasis was the most common among these tumors.

These findings reinforce the critical role of histopathology in establishing a definitive diagnosis and highlight the importance of anatomical localization in determining the symptomatology. They also support ongoing efforts to refine the diagnostic and therapeutic strategies for this heterogeneous and clinically significant group of neoplasms.

## 5. Study Limitations

Although extensive, this study had several limitations to consider when interpreting the findings. The retrospective nature of this study limits our ability to ascertain causality between clinical presentations and tumor characteristics and may introduce recall or selection bias. The study included only patients who underwent surgery with histopathologically confirmed diagnoses, excluding those with inoperable tumors or those managed non-surgically. This may have underestimated the prevalence of more aggressive or advanced tumors. This study lacks comprehensive follow-up data on long-term outcomes after diagnosis and treatment, limiting our understanding of the prognostic implications for various cardiac tumor types. While echocardiography was primarily used, the adoption of other imaging modalities, such as MRI and CT, was not uniform across cases, potentially affecting the consistency in tumor detection and characterization. Future studies should address these limitations through a prospective cohort design, broader multinational participation, inclusion of non-surgically managed cases, longitudinal follow-up, and standardized diagnostic protocols across centers to enhance the reliability of comparisons across patient groups.

## 6. Conclusions

The findings from our comprehensive analysis confirm that while the majority of heart tumors exhibit benign histology, their potential to interfere with cardiac mechanical and electrical functions is significant, often leading to severe clinical outcomes. Diagnostic imaging, particularly echocardiography, plays a pivotal role in the initial detection and evaluation of these tumors. However, appropriate management strategies are predominantly dependent on histopathological examination.

This study’s reliance on and analysis of histopathologically confirmed cases provide robust insights into the nature and implications of cardiac tumors. In doing so, it makes a substantial contribution to the existing medical literature. Another key finding of our study is that, even though the 5th edition of the 2021 WHO Classification of Thoracic Tumors lists PFE as the most common primary cardiac tumor, myxoma is actually the most common primary cardiac tumor in our study and in many other studies. This finding demonstrates a significant discrepancy between the current international classification and real-world data and suggests that tumor distribution may be related to regional and demographic differences.

## Figures and Tables

**Table 1:** Descriptive characteristics of cardiac tumors.

Characteristic	*n*	%
**Gender**		
Male	120	43.8
Female	154	56.2
**Symptoms**		
Asymptomatic	17	23.9
Cardiac Symptoms	51	71.8
- Dyspnea	31	43.7
- Tachycardia	3	4.2
- Chest Pain	16	22.5
- Palpitations	12	16.9
- Other Cardiac Symptoms	4	5.6
Embolic Symptoms	10	14.1
Systemic Symptoms	15	21.1
**Complications**		
- Infection	67	94.4
- Effusion	60	84.5
- Arrhythmia	61	85.9
**Comorbidities**		
- Coronary Artery Disease	18	20.2
- Diabetes Mellitus	11	12.4
- Hypertension	10	11.2
- Neuropsychiatric Disorders	4	4.5
- Known Malignancy	13	14.6
**Imaging Findings**		
Echocardiographic Findings	51	57.3
**Other Imaging**		
- MRI	4	4.5
- CT	3	1.1
- None	82	92.1

MRI: magnetic resonance imaging, CT: computed tomography.

**Table 2:** Distribution of pathological diagnoses of cardiac tumors.

Diagnosis	*N*	%
**Primary Benign Tumors**		
Myxoma	192	70.1
Papillary Fibroelastoma	28	10.2
Vascular Benign Tumor	5	1.82
Other Benign Tumor	11	4.01
**Primary Malignant Tumors**		
Sarcoma	8	2.92
Vascular Malignant Tumor	5	1.82
Other Malignant Tumor *	6	2.2
**Metastatic Tumor**	19	6.93
**Total**	**274**	**100.0**

* Haematolymphoid malignancies of the heart, small-round-cell-tumor, leiomyosarcoma, and undifferentiated pleomorphic sarcoma.

**Table 3:** Distribution of pathological diagnoses by tumor location.

Tumor Location	Pathological Diagnosis	*N*	%
Left Atrium (67 cases)	Myxoma	66	98.5
	Sarcoma	1	1.5
Right Atrium (11 cases)	Myxoma	6	50.0
	Metastatic Tumor	2	16.7
	Angiosarcoma	1	8.3
	Sarcoma	1	8.3
	Small Round Cell Tumor	1	8.3
Mitral Valve (6 cases)	Myxoma	2	40.0
	Papillary Fibroelastoma	4	60.0
Tricuspid Valve (5 cases)	Myxoma	1	20.0
	Papillary Fibroelastoma	4	80.0
Left Ventricle (2 cases)	Papillary Fibroelastoma	1	50.0
	Vascular Benign Tumor	1	50.0
Right Ventricle (1 case)	Metastatic Tumor	1	100.0
Pericardium (9 cases)	Metastatic Tumor	9	100.0
Pulmonary Valve (1 case)	Papillary Fibroelastoma	1	100.0
Aortic Valve (1 case)	Papillary Fibroelastoma	1	100.0
Pulmonary Artery and Aortic Valve (1 case)	Sarcoma	1	100.0
Unspecified Location (12 cases)	Myxoma	12	100.0

**Table 4:** Comparison of tumor size and age according to symptom status.

Variable	Symptomatic (*n* = 52)	Asymptomatic (*n* = 17)	*p*-Value
Age (years)	53.38 ± 16.25 (median: 56.5)	58.71 ± 14.09 (median: 62.0)	0.23
Tumor Size (mm)	30.25 ± 20.57	39.07 ± 19.69	0.14

## Data Availability

The raw data supporting the conclusions of this article will be made available by the authors on request.
